# Simultan-integrierter Boost bei moderat hypofraktionierter adjuvanter Radiotherapie der Brust

**DOI:** 10.1007/s00066-023-02144-z

**Published:** 2023-09-12

**Authors:** David Krug, Jürgen Dunst

**Affiliations:** Klinik für Strahlentherapie/Radioonkologie, UKSH, Campus Kiel, Kiel, Deutschland

## Hintergrund

Die moderat hypofraktionierte Radiotherapie hat die konventionelle Fraktionierung als Standard bezüglich der adjuvanten Radiotherapie der Brust abgelöst. In den relevanten randomisiert-kontrollierten Studien wurde jedoch entweder kein Boost oder ein sequenzieller Boost in konventioneller Fraktionierung mit 5–8 × 2 Gy verabreicht. Während der simultan-integrierte Boost (SIB) bei konventioneller Fraktionierung bereits routinemäßig zum Einsatz kam, lagen bei moderater Fraktionierung bislang nur einarmige Phase-II-Studien und kleine randomisiert-kontrollierte Studien vor.

## Patienten und Methodik

Die IMPORT-HIGH-Studie wurde als Nichtunterlegenheitsstudie konzipiert. Eingeschlossen werden konnten Patientinnen mit invasivem Mammakarzinom im Stadium pT1–3 pN0–3a nach brusterhaltender Operation mit R0-Resektion. Das Design ist analog zur IMPORT-LOW-Studie. In den beiden experimentellen Armen wurden drei Subvolumina der zu bestrahlenden operierten Brust behandelt. Einerseits bestanden diese aus einem „clinical target volume“ (CTV) für den Boost, das das Tumorbett inkl. Clips und postoperativer Veränderungen einschloss. Dieses wurde um 5 mm zum „boost-planning target volume“ (PTV) erweitert. Es wurde weiterhin ein Teilbrust-CTV definiert, das das Boost-CTV mit einem Sicherheitsabstand von 15 mm einschloss. Dieses wurde um 10 mm zum Teilbrust-PTV erweitert. Weiterhin wurde die operierte Brust außerhalb der genannten Subvolumina behandelt.

Im Standardarm wurde die operierte Brust mit 40 Gy in 15 Fraktionen und einem sequenziellen Boost auf das Boost-PTV von 8 × 2 Gy bestrahlt. Ähnlich wie in der IMPORT-LOW-Studie standen dem zwei experimentelle Arme gegenüber. In beiden experimentellen Armen erhielten die Patientinnen eine Bestrahlung mit 36 Gy (15 × 2,4 Gy) auf die gesamte Brust sowie mit 40 Gy (15 × 2,67 Gy) auf das Teilbrust-PTV. In „test group 1“ (im Weiteren als „48 Gy-Arm“ bezeichnet) wurden auf das Boost-PTV 48 Gy (15 × 3,2 Gy) verabreicht, in „test group 2“ (im Weiteren als „53 Gy-Arm“ bezeichnet) waren es 53 Gy (15 × 3,53 Gy). Primärer Endpunkt war die Lokalrezidivrate nach 5 Jahren. Es wurde eine Lokalrezidivrate von 5 % im Standardarm angenommen. Die Nichtunterlegenheit wurde als ein Anstieg der Lokalrezidivrate in den experimentellen Armen um ≤ 3 % definiert (entsprechend einer Hazard Ratio [HR] = 1,63). Sekundäre Endpunkte waren die Rezidivlokalisation, regionäre Rezidive, Fernmetastasierung, krankheitsfreies Überleben, Gesamtüberleben und Spättoxizität. Die Akuttoxizität wurde nicht untersucht. In Substudien wurden patientenberichtete Endpunkte erhoben und eine Fotodokumentation durchgeführt. Für die Toxizitätsendpunkte wurde aufgrund multiplen Testens ein *p*-Wert von 0,01 als Signifikanzniveau definiert.

## Ergebnisse

Es wurden zwischen 2009 und 2015 insgesamt 2617 Patientinnen eingeschlossen und 1:1:1 auf die drei Arme randomisiert. Die mediane Nachbeobachtungszeit betrug 74 Monate. Die Lokalrezidivraten nach 5 Jahren betrugen 1,9 % im Standardarm, 2,0 % im 48 Gy-Arm und 3,2 % im 53 Gy-Arm. Bezüglich der absoluten Unterschiede in den Lokalrezidivraten konnte die Nichtunterlegenheit für den 48 Gy-Arm gezeigt werden (absoluter Unterschied +0,1 %; 95 %-Konfidenzintervall [KI] −0,8 bis +1,7 %; HR 1,04; 95 %-KI 0,56–1,92), nicht jedoch für den 53 Gy-Arm (absoluter Unterschied +1,4 %; 95 %-KI −0,03 bis +3,8 %; HR 1,76; 95 %-KI 1,02–3,04). Relativ betrachtet konnte die Nichtunterlegenheit jedoch für beide Arme nicht gezeigt werden, da die Konfidenzintervalle für beide experimentellen Arme die präspezifizierte Hazard Ratio von 1,63 miteinschlossen. Die Lokalrezidive waren zu 44,7 % im Boost-PTV und zu 15,8 % im Teilbrust-PTV (außerhalb des Boost-PTV) lokalisiert. In den sonstigen onkologischen Endpunkten gab es keine signifikanten Unterschiede. Die Langzeitmorbidität fiel im 48 Gy-Arm am günstigsten aus. Die Rate an mäßigen oder ausgeprägten Veränderungen in der Brust unterschied sich nicht signifikant zwischen dem Standardarm und den beiden experimentellen Armen, war jedoch im 53 Gy-Arm höher als im 48 Gy-Arm (34,5 % vs. 25,8 %; *p* = 0,026). Erhöhte Nebenwirkungsraten zwischen dem 48 Gy-Arm und dem 53 Gy-Arm zeigten sich ebenfalls für Formveränderungen der Brust (*p* = 0,0085), Induration im Boostquadranten (*p* = 0,0005), Druckschmerzhaftigkeit (*p* = 0,014) und Brustschmerzen (*p* = 0,0025). Ein Brustödem trat im 48 Gy-Arm signifikant seltener auf als im Kontrollarm (*p* = 0,0062). Brustschmerzen (*p* = 0,0081) und Induration (*p* = 0,015) im Boostquadranten waren im 53 Gy-Arm häufiger vorhanden als im Standardarm. Die weiteren Auswertungen der Spättoxizität inkl. der patientenberichteten Endpunkte bestätigten die insgesamt vergleichbaren Ergebnisse zwischen den Behandlungsarmen mit günstigerem Toxizitätsprofil im 48 Gy-Arm. In der Auswertung der fotografischen Dokumentation zeigte sich ein selteneres Auftreten von milden bis ausgeprägten Brustveränderungen im 48 Gy-Arm verglichen mit dem Standardarm (*p* = 0,021).

## Bewertung der Autoren

Die moderat hypofraktionierte Radiotherapie der Brust nach brusterhaltender Operation ist sicher und reduziert die Anzahl der Behandlungssitzungen. Eine Eskalation der Boostdosis ist nicht zu empfehlen.

## Kommentar

Die moderat hypofraktionierte Radiotherapie ist der klare Standard in der Fraktionierung der adjuvanten Radiotherapie nach brusterhaltender Therapie [1]. Dass bislang nur eine eingeschränkte Datenlage für den SIB bei moderater Hypofraktionierung bestand und somit die Verabreichung eines sequenziellen Boosts empfohlen wurde, wurde mitunter als Argument für die konventionelle Fraktionierung genutzt. Gut zu beobachten ist diese klinische Praxis in den Daten der HYPOSIB-Studie [2], in der im Standardarm gemäß der Entscheidung in den Studienzentren vor Ort 55,3 % der Patientinnen eine konventionell fraktionierte Radiotherapie mit SIB erhielten und 33,8 % der Patientinnen eine moderate Hypofraktionierung mit sequenziellem Boost – obwohl randomisiert-kontrollierte Studien für die konventionelle Fraktionierung mit SIB erst nach Ende der Rekrutierung publiziert wurden [3, 4]. Im Verlauf der Rekrutierung wurde die moderate Hypofraktionierung mit sequenziellem Boost zum am häufigsten verwendeten Schema im Standardarm, was vor allem durch einen vermehrten Einsatz an den universitären Studienzentren bedingt war [2].

Mehrere Aspekte der IMPORT-HIGH-Studie sind interessant und bedürfen einer weiteren Diskussion:

1. Die Reduktion der Dosis im Brustvolumen außerhalb des Teilbrust-PTV von 40 auf 36 Gy führte ähnlich wie in der IMPORT-LOW-Studie zu einer tendenziell günstigeren Langzeitmorbidität im Vergleich zum Standardarm. In beiden Studien zeigten sich keine erhöhten Rezidivraten außerhalb des Teilbrust-PTV. Da außerhalb des Teilbrust-PTV wahrscheinlich eher Zweitkarzinome als „echte“ Lokalrezidive zu erwarten sind und die zeitliche Latenz hier deutlich größer ist, sollten die Langzeitergebnisse abgewartet werden. In den Konkurrenzstudien RTOG 1005 und HYPOSIB wurden jeweils 40 Gy in 15 bzw. 16 Fraktionen auf das Brustparenchym außerhalb des Boostvolumens verabreicht.
2. Die Eskalation der Boostdosis von 48 Gy (EQD2Gy_α/β_ _=_ _3,5_ _Gy_ = 58,5 Gy) auf 53 Gy (EQD2Gy_α/β_ _=_ _3,5_ _Gy_ = 67,7 Gy) im SIB-PTV führte zu einem höheren Lokalrezidivrisiko und einer vermehrten Spättoxizität. Auch wenn die Erhöhung der Lokalrezidivrate rational nicht zu erklären ist, fällt die Nutzen-Risiko-Bilanz klar negativ aus. Was die optimale Dosis im Boostvolumen ist, bleibt weiter unklar. Einen randomisierten Vergleich zwischen den am häufigsten eingesetzten sequenziellen Boostschemata von 5 × 2 und 8 × 2 Gy gibt es nicht. In einer Substudie der EORTC-Studie konnte bei R1-Resektion kein Vorteil einer Dosiseskalation von 5 × 2 Gy auf 13 × 2 Gy gezeigt werden [5]. Die Effektivitätsdaten der Young-Boost-Studie, in der 8 × 2 Gy und 13 × 2 Gy verglichen wurden, sind weiter ausstehend. In beiden Dosiseskalationsstudien wurden jedoch signifikant höhere Fibroseraten und in der Young-Boost-Studie weiterhin ein schlechteres kosmetisches Ergebnis berichtet [5, 6]. Eine Dosiseskalation im Boostvolumen sollte also außerhalb klinischer Studien nicht zum Einsatz kommen.
3. Die Boostvolumina waren in der IMPORT-HIGH-Studie mit einem medianen Volumen von 12,5 bis 13,5 cm^3^ sehr klein. Zum Vergleich: In der Young-Boost-Studie lag das mediane Boostvolumen bei 132 cm^3^ [6]. Es wurden in der IMPORT-HIGH-Studie ausnahmslos Photonen verwendet. In 45,7 % wurde mit inverser Optimierung geplant, die restlichen Fälle verteilten sich etwa hälftig auf vorwärts geplante Feld-in-Feld-Tangenten und Hybridtechniken. Der Einsatz von Clips zur Definition des Boostvolumens war zwar nicht obligat, aber empfohlen, und Clips wurden in > 80 % der Zentren routinemäßig implantiert [7]. Eine dementsprechende Empfehlung wurde auch in den diesjährigen AGO-Leitlinien implementiert [8]. Weiterhin kam eine bildgesteuerte Therapie zum Einsatz. Die Reduktion des Boostvolumens wurde in allen Behandlungsarmen angewendet. Ein negativer Einfluss auf die Behandlungsergebnisse war nicht offenkundig zu beobachten. Die Lokalrezidivraten fallen trotz der negativen Patientenselektion (medianes Alter < 50 Jahren, 65 % adjuvante Chemotherapie, > 50 % G3, ca. 30 % Nodalbefall) günstig aus.

Wo geht die Reise hin für die Boostbestrahlung? Die Daten der IMPORT-HIGH-Studie bieten gemeinsam mit den bei der ASTRO-Jahrestagung 2022 präsentierten Daten der RTOG-1005-Studie [9] eine sehr gute Grundlage für den Einsatz eines SIB bei moderat hypofraktionierter Radiotherapie der Brust [8]. Die Effektivitätsdaten der HYPOSIB-Studie sind 2024 zu erwarten, hinsichtlich der Akuttoxizität zeigten sich bereits reduzierte Radiodermatitisraten für die moderate Hypofraktionierung mit SIB [10]. Wie in vielen Studien zu beobachten ist, nehmen die Lokalrezidivraten deutlich ab und liegen in der Regel bei 1–2 % nach 5 Jahren. Dies trifft auch für die Patientinnen mit Niedrigrisikomammakarzinom zu. Interessant ist, dass die Indikationsstellung für die Boostbestrahlung international deutliche Variationen aufweist. In der DBCG-HYPO-Studie beispielsweise erhielten in den dänischen und norwegischen Studienzentren 0–15 % der Patientinnen eine Boostbestrahlung, während es in den deutschen Studienzentren 85 % der Patientinnen waren [11]. Aufgrund der signifikanten Erhöhung des Fibroserisikos sollte die Boostindikation bei Patientinnen > 50 Jahre nur bei vorhandenen Risikofaktoren gestellt werden. Studien zur Ultrahypofraktionierung mit SIB laufen bereits.

*David Krug und Jürgen Dunst, Kiel*
